# IL-3 Is a Marker of Encephalitogenic T Cells, but Not Essential for CNS Autoimmunity

**DOI:** 10.3389/fimmu.2018.01255

**Published:** 2018-06-04

**Authors:** Priscilla W. Lee, Matthew K. Xin, Wei Pei, Yuhong Yang, Amy E. Lovett-Racke

**Affiliations:** ^1^Department of Microbial Infection and Immunity, The Ohio State University, Columbus, OH, United States; ^2^Department of Neurology, The Ohio State University, Columbus, OH, United States

**Keywords:** multiple sclerosis, experimental autoimmune encephalomyelitis, IL-3, GM-CSF, Th1 cells, Th17 cells

## Abstract

Identifying molecules that are differentially expressed in encephalitogenic T cells is critical to the development of novel and specific therapies for multiple sclerosis (MS). In this study, IL-3 was identified as a molecule highly expressed in encephalitogenic Th1 and Th17 cells, but not in myelin-specific non-encephalitogenic Th1 and Th17 cells. However, B10.PL IL-3-deficient mice remained susceptible to experimental autoimmune encephalomyelitis (EAE), a mouse model of MS. Furthermore, B10.PL myelin-specific T cell receptor transgenic IL-3^−/−^ Th1 and Th17 cells were capable of transferring EAE to wild-type mice. Antibody neutralization of IL-3 produced by encephalitogenic Th1 and Th17 cells failed to alter their ability to transfer EAE. Thus, IL-3 is highly expressed in myelin-specific T cells capable of inducing EAE compared to activated, non-encephalitogenic myelin-specific T cells. However, loss of IL-3 in encephalitogenic T cells does not reduce their pathogenicity, indicating that IL-3 is a marker of encephalitogenic T cells, but not a critical element in their pathogenic capacity.

## Introduction

Multiple sclerosis (MS) is a CNS demyelinating disease that is postulated to be mediated by encephalitogenic CD4^+^ T cells reactive to myelin (1). Most individuals harbor myelin-reactive T cells, illustrating that the mere presence of myelin-specific T cells is insufficient to cause MS (2–4). It is still unclear what characteristics of a myelin-specific T cell make them encephalitogenic and capable of mediating CNS pathology. Identifying molecules that are differentially expressed in encephalitogenic T cells is critical to understanding how they mediate pathology, as well as identifying new and specific therapeutic targets for MS. IL-3 is a molecule that is primarily produced by activated T-cells (5), but plays a critical role in the activation and survival of a diverse group of cells, including mast cells and basophils, monocytes, B cells, T cells, and endothelial cells (6–13). Studies have shown that IL-3 contributes to inflammation and autoimmunity (14–16). The *Il3* gene is located on chromosome 11 in mice and chromosome 5 in humans, within the same gene cluster as *Csf2*, the gene coding GM-CSF. There is a potential binding site for the transcriptional factor T-bet close to this gene cluster. T-bet and GM-CSF have been shown to be associated with EAE development (17–24), and so it is possible that the activation of this gene cluster is a contributing factor to T cell encephalitogenicity. A previous study found that increased IL-3 expression by CD4^+^ T cells is associated with relapses in MS patients, encephalitogenic CD4^+^ T cells are the primary source of IL-3 in EAE, and the administration of IL-3 to mice with EAE exacerbates disease (16, 25). IL-3 has also been identified as a signature gene of pathogenic Th17 cells in EAE (26), and IL-3 was shown to be higher in myelin tetramer-positive memory T cells from MS patients (27). In this study, we analyze the role of IL-3 produced by T cells in EAE using B10.PL mice and myelin-specific T cell receptor (TCR) transgenic cells, which allows us to efficiently generate encephalitogenic and non-encephalitogenic T cells with a single *in vitro* stimulation so that we can compare the role of IL-3 in T cell encephalitogenicity (28).

## Materials and Methods

### Animals

IL-3^−/−^ mice (29) were backcrossed >8 generations onto the B10.PL background and MBP Ac1-11-specific TCR transgenic mice (30). The protocols used for these experiments received approval by the OSU Institutional Animal Care and Use Committee and were conducted in accordance with the US Public Health Service’s Policy on Humane Care and Use of Laboratory Animals.

### *In Vitro* Culture of Splenocytes

Splenocytes were isolated from 6- to 8-week TCR transgenic mice and cultured in 24-well plates at 1.5 × 10^6^ cells/well with irradiated splenocytes (feeder cells) from wild-type mice at a concentration of 4.5 × 10^6^ cells/well and MBP Ac1-11 peptide (2 µg/mL). Th17 cells were induced by adding IL-6 (25 ng/mL) plus anti-IL4 (1 μg/mL; 30340), anti-IL12 (0.5 µg/mL; polyclonal goat IgG), and anti-IFNγ (2 μg/mL; H22) to the cultures or IL-6 + TGFβ (1 ng/mL). Th1 cells were induced by adding IL-12 (0.5 ng/mL) to the cultures or activating the T cells with anti-CD3/CD28 (145-2C11 and 37.51, BD Biosciences, San Jose, CA, USA)-coated plates plus IL-12 (0.5 ng/mL). Antibodies and cytokines were purchased from R&D Systems (Minneapolis, MN, USA). Some cells were treated with IL-3-neutralizing antibody (2 μg/mL; MP2-8F8, BioLegend, San Diego, CA, USA).

### ELISA

The following antibodies were used: IL-3 capture (MP2-8F8) and biotinylated IL-3 detection (MP2-43D11) (BioLegend), IL-17 capture (eBio17CK15A5) and biotinylated IL-17 detection (eBio17B7) (eBioscience, Waltham, MA, USA), IFNγ capture (R46A2) and biotinylated IFNγ detection (XMG1.2) (BD Biosciences), and GM-CSF capture (MP122E9) and biotinylated GM-CSF detection (polyclonal goat IgG) (R&D Systems) were used. The ELISA was performed as previously described (30).

### EAE Induction

Active induction of EAE was performed by subcutaneous injection of naïve 6–8 week B10.PL IL-3^−/−^, IL-3^+/−^, and IL-3^+/+^ littermate mice over four sites in the flanks with 200 µg MBP Ac1-11 (CS Bio, Menlo Park, CA, USA) emulsed in CFA (Difco, Becton Dickinson Co., Franklin Lakes, NJ, USA). Pertussis toxin (200 ng) (List Biological Laboratories, Campbell, CA, USA) was injected i.p. at the time of immunization and 48 h later. For adoptive transfer EAE, splenocytes were isolated from 6- to 8-week IL-3^−/−^, IL-3^+/−^, and IL-3^+/+^ TCR transgenic mice. Cells were activated *in vitro* as described above, collected at 72 h, and 5 × 10^6^ cells were injected i.p. into B10.PL mice. Mice were evaluated daily for signs of EAE: 0, no clinical disease; 1, limp/flaccid tail; 2, moderate hind limb weakness; 3, severe hind limb weakness; 4, complete hind limb paralysis; 5 quadriplegia or premoribund state; and 6, death.

### Flow Cytometry

Cells were collected, washed, and resuspended in staining buffer (1% BSA in PBS) and incubated with Fc blocker (93, BioLegend) for 10 min at 4°C. Cells were then stained for cell-surface markers for 30 min at 4°C. After washing twice with buffer, cells were fixed and permeabilized using Cytofix/Cytoperm solution (BD Biosciences) for 20 min at 4°C. Cells were stained for intracellular cytokines with Ab diluted in Perm/Wash solution (BD Biosciences) for 30 min at 4°C. After washing twice with Perm/Wash buffer, the cells were resuspended in 200 µL of buffer. Approximately 100,000 cell events were acquired on a FACSCanto II (BD Biosciences) and analyzed using FlowJo software (Tree Star, Inc., Ashland, OH, USA). Pacific Blue-anti-CD4 (RM4-5), FITC-anti-CD44 (IM7), PE-anti-IL-3 (MP2-8F8), PE-anti-GM-CSF (MP1-22E9), APC-anti-IFNγ (XMG1.2), and PE-anti-IL-17 (TC11-18H10) were obtained from BD Bioscience.

## Results

**Figure 1:**
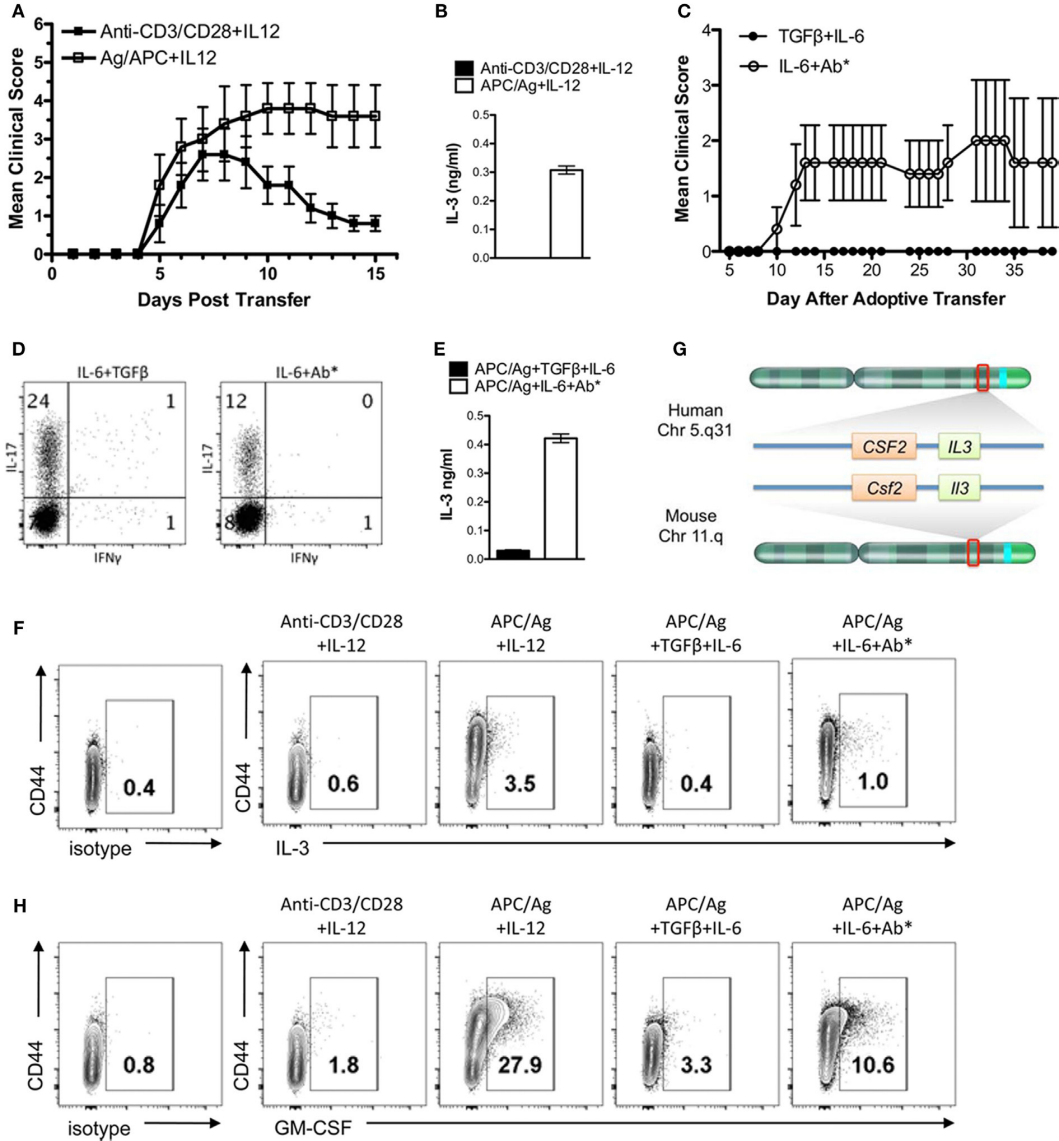
IL-3 is highly expressed in encephalitogenic Th1 and Th17 cells. Splenocytes from naïve MBP-specific T cell receptor Tg mice were differentiated into Th1 cells with plate-bound anti-CD3/28 Ab plus IL-12 or antigen-presenting cell (APC)/Ag plus IL-12. Th17 cells were differentiated with APC/Ag plus IL-6 + TGF-β or APC/Ag plus IL-6 + anti-IL4/IL12/IFNγ. **(A,C)** Cells were collected and transferred into B10.PL mice (5 × 10^6^ cells/mouse). Experimental autoimmune encephalomyelitis clinical scores were monitored daily. Data are representative of three independent experiments (mean ± SEM). **p < 0.01, Mann–Whitney *U* test. **(B,E)** Supernatants were analyzed by ELISA for IL-3. **(D)** Flow cytometric analysis of IL-17 and IFNγ in Th17 cells. **(F)** IL-3 and **(H)** GM-CSF were stained intracellularly and analyzed by flow cytometry (gated on CD4^+^ cells). **(G)** Illustration of IL-3 and GM-CSF loci in mouse and human chromosomes.

The encephalitogenicity of myelin-specific T cells varies using different *in vitro* activation protocols. In Figure 1A, naïve myelin-specific T cells from a MBP Ac1-11-specific TCR transgenic mouse were differentiated with anti-CD3/CD28 + IL-12 or APCs + MBP Ac1-11 peptide + IL-12 to generate myelin-specific Th1 cells. Th1 cells differentiated with APC/Ag + IL-12 were highly encephalitogenic, while the myelin-specific Th1 cells differentiated with anti-CD3/CD28 + IL-12 were significantly less encephalitogenic. Analysis of IL-3 by these Th1 cells found that IL-3 expression was significantly elevated in these APC/Ag-driven Th1 cells (Figure 1B). Similarly, myelin-specific Th17 cells differentiated with APC/Ag + IL-6 + TGFβ were not encephalitogenic, while differentiation of MBP-specific TCR transgenic T cells into Th17 with APC/Ag + IL-6 + anti-IL4/IL12/IFNγ were highly encephalitogenic (Figures 1C,D), consistent with previous studies (22, 28, 31–34). Similar to the encephalitogenic Th1 cells, IL-3 was highly expressed in the encephalitogenic Th17 cells (Figure 1E). The percentage of IL-3^+^ cells in the activated CD4^+^ T cell population was enhanced in the encephalitogenic Th1 and Th17 cells (Figure 1F). Since the *Il3* allele is located in the same gene cluster with *Csf2* (encodes GM-CSF) on chromosome 11 in mouse and chromosome 5 in human (Figure 1G) and GM-CSF was found to be critical for encephalitogenic T cells (17, 21, 23, 24), we analyzed GM-CSF^+^-activated CD4 T cells *via* flow cytometry. As expected, GM-CSF^+^ cells were increased in encephalitogenic Th1 and Th17 cells (Figure 1H). Given that *Il3* and *Csf2* are in the same gene cluster, it is unclear whether increased IL-3 is due to a specific upregulation of the *Il3* gene or an indirect effect due to upregulation of the *Il3*/*Csf2* gene cluster.

**Figure 2:**
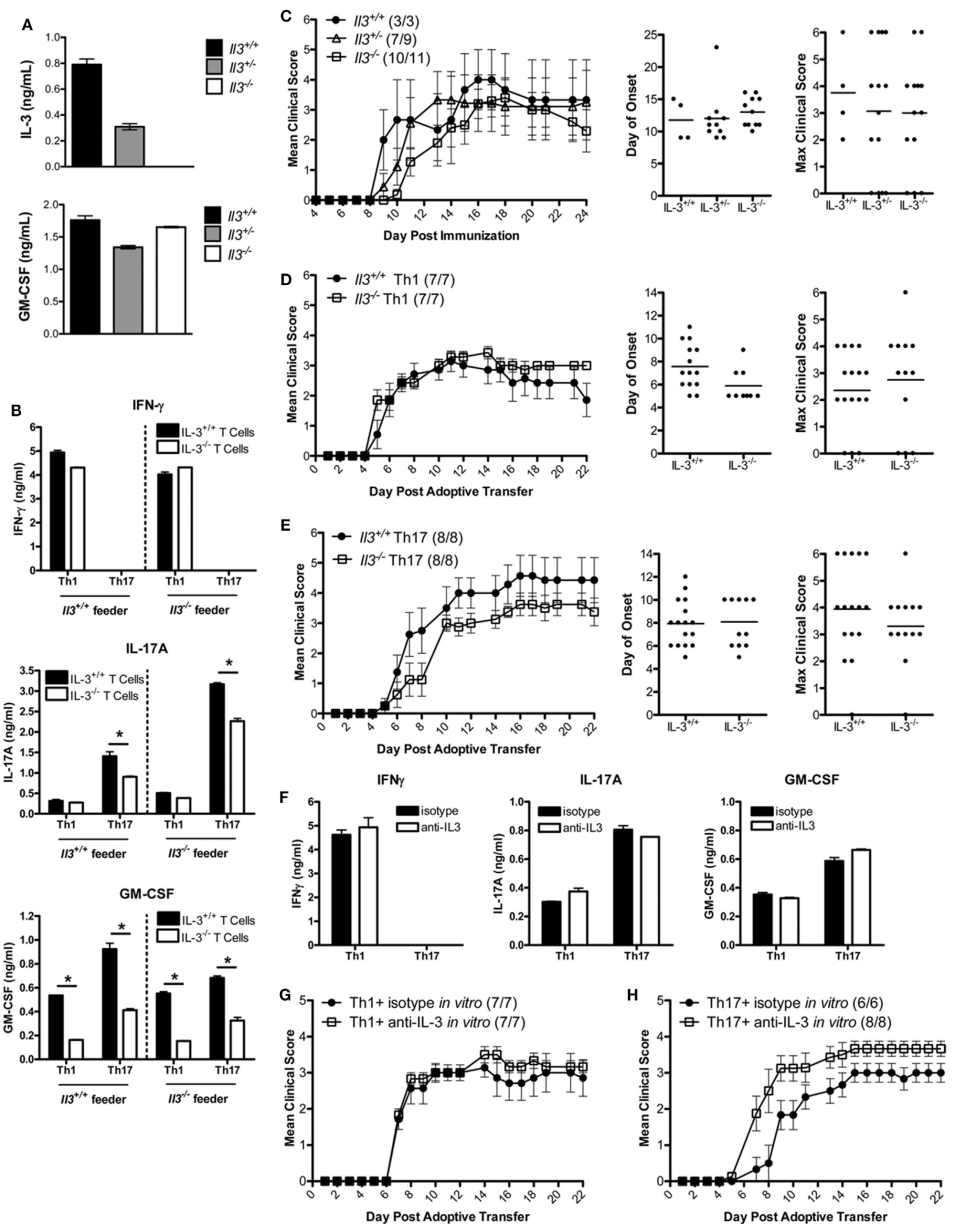
IL-3 is not required for T cell encephalitogenicity. **(A)** Splenocytes from naïve *Il3^+/+^, Il3*^+/−^, and *Il3*^−^*^/^*^−^ MBP-specific T cell receptor (TCR) Tg mice were activated with MBP Ac1-11 and feeder cells without exogenous cytokines *in vitro* for 3 days. Supernatants were collected and analyzed by ELISA for IL-3 and GM-CSF. **(B)** *Il3^+/+^* or *Il3*^−^*^/^*^−^ TCR Tg splenocytes were cocultured with *Il3^+/+^* or *Il3*^−^*^/^*^−^ feeder cells in the presence of MBP Ac1-11 peptide *in vitro* for 3 days. Encephalitogenic Th1 cells were differentiated with IL-12, and encephalitogenic Th17 cells were differentiated with IL-6 + anti-IL4/IL12/IFNγ. Supernatants were analyzed by ELISA for IFNγ, IL-17A, and GM-CSF. *p < 0.05, unpaired Student’s *t*-test. **(C)** Experimental autoimmune encephalomyelitis (EAE) was induced by immunization of B10.PL *Il3^+/+^ Il3*^+/−^ and *Il3*^−^*^/^*^−^ mice with MBP Ac1-11/CFA. Disease incidence is in parentheses. Day of disease onset and maximum clinical scores for each mouse are shown for two experiments. **(D,E)** Splenocytes from naïve *Il3^+/+^* and *Il3*^−^*^/^*^−^ TCR Tg mice were activated with MBP Ac1-11 peptide under encephalitogenic Th1 and Th17 conditions for 3 days. **(D)** Th1 cells and **(E)** Th17 cells were collected and adoptively transferred into B10.PL mice (5 × 10^6^ cells/mouse). EAE clinical scores were monitored daily (mean ± SEM). Day of disease onset and maximum clinical scores for each mouse are shown for all replicate experiments. **(F–H)** Splenocytes from naïve MBP-specific TCR Tg mice were activated with MBP Ac1-11 peptide under encephalitogenic Th1 and Th17 conditions in the presence of anti-IL-3 or isotype Ab for 3 days. **(F)** Supernatants were analyzed by ELISA for IFNγ, IL-17A, and GM-CSF. **(G)** Th1 cells and **(H)** Th17 cells were collected and adoptively transferred into naive B10.PL mice (5 × 10^6^ cells per mouse).

To determine whether IL-3 was critical in the encephalitogenic capacity of myelin-specific T cells, IL-3-deficient mice were backcrossed with the B10.PL MBP-specific TCR transgenic mice. Since the *Il3* and *Csf2* alleles are in close proximity, we needed to confirm that deletion of *Il3* gene did not result in a loss of *Csf2* gene expression. IL-3^+/+^, IL-3^+/−^, and IL-3^−/−^ splenocytes were activated with MBP Ac1-11 *in vitro*, and IL-3 and GM-CSF levels were analyzed by ELISA. GM-CSF levels were not significantly changed in the IL-3^+/−^ or IL-3^−/−^ splenocytes, indicating that *Csf2* gene expression was intact (Figure 2A). To determine if IL-3 deficiency influenced Th1 and Th17 cell differentiation, the myelin-specific T cells were differentiated under Th1 and Th17 conditions *in vitro* and analyzed for cytokine production by ELISA. Since APCs also produce IL-3, crisscross cultures were set up with myelin-specific IL-3^+/+^ and IL-3^−/−^ T cells cocultured with IL-3^+/+^ and IL-3^−/−^ APCs (feeder cells) under encephalitogenic Th1 and Th17 conditions (Figure 2B). For Th1 cells, IFNγ levels were not significantly changed, but there was a reduction in GM-CSF levels. There was no difference between Th1 cells cultured with IL-3^+/+^ or IL-3^−/−^ APCs. There was a reduction in IL-17 and GM-CSF levels in the Th17 cells, and surprisingly IL-17 expression was higher with IL-3^−/−^ APCs. The reduction in GM-CSF in both the Th1 and Th17 cells was suggestive of reduced encephalitogenicity since GM-CSF was found to be essential in encephalitogenic T cells.

To determine if IL-3 deficiency protects mice from EAE development, B10.PL IL-3^+/+^, IL-3^+/−^, and IL-3^−/−^ mice were immunized with MBP Ac1-11/CFA. The incidence, day of onset, disease course, and maximum clinical course were not significantly different between the groups (Figure 2C). To specifically address the role of IL-3 in CD4 T cells, myelin-specific IL-3^+/+^ and IL-3^−/−^ T cells were differentiated into encephalitogenic Th1 and Th17 cells and transferred into B10.PL mice. The incidence of disease, day of onset, and maximum clinical score were not significantly different between the mice that received the IL-3^+/+^ and IL-3^−/−^ T cells (Figures 2D,E). There was also no difference in disease course between IL-3^+/+^ and IL-3^−/−^ Th1 cells (Figure 2D). There was a slight decrease in disease course in the IL-3^−/−^ Th17 cells (Figure 2E), but this was not reproducible.

To validate these data, myelin-specific CD4 T cells were cultured with a neutralizing IL-3 antibody during the *in vitro* differentiation into encephalitogenic Th1 and Th17 cells. Although there was a complete abrogation of IL-3 with the antibody (data not shown), there was no change in IFNγ, IL-17, or GM-CSF expression (Figure 2F). There was also no reduction in the encephalitogenicity of myelin-specific Th1 or Th17 cells with IL-3 neutralization (Figures 2G,H), indicating that IL-3 was not essential for the generation of encephalitogenic T cells. These data suggest that IL-3 levels are associated with the encephalitogenic capacity of myelin-specific Th1 and Th17 cells, but IL-3 plays no major role in the encephalitogenic capacity of the cells.

## Discussion

As the role of IL-3 in MS is not well-described, the goal of this study was to determine if myelin-specific IL-3 T cell production was necessary for encephalitogenicity. IL-3 expression was significantly higher on encephalitogenic T cells, but loss of IL-3 in T cells had no significant effect on the development or severity of EAE. Previous studies have demonstrated that IL-3 is highly expressed in encephalitogenic cells (16, 25, 26), consistent with the data from this study. However, the high expression of IL-3 in combination with the high expression of GM-CSF, a cytokine located in the same gene cluster, indicates that the upregulation of these two genes may be a product of increased upstream promotor function and not as a causative factor in encephalitogenicity. Renner et al. (16) have shown results contradictory to the findings in this study, which may be due to differences in experimental design. Their study found a modest reduction in B6/MOG EAE severity at days 18–20 using a parametric *t*-test, instead of analyzing EAE using a non-parametric test since the EAE scoring system is not linear. Their study also found that EAE was more severe in IL-3^−/−^ mice than IL-3^+/−^ mice, which is inconsistent with their conclusion. However, they did observe that administration of IL-3 *in vivo* enhanced EAE severity and administration of anti-IL3 modestly lessened disease severity, indicating that IL-3 may contribute to pathology.

IL-3 has been implicated as both a pro-inflammatory and anti-inflammatory cytokine. IL-3 has been shown to not only contribute to microglia activation (35) but also protect neurons from inflammation following mechanical strain (36). IL-3 transcripts have been shown to be upregulated in CNS lesions of MS patients (37), and chronic expression of IL-3 by astrocytes results in an MS-like disease in mice (38). In the present study, we differentiated the effects of systemic loss of IL-3 compared to T cell-specific loss of IL-3 and found that IL-3 was not a major contributor to the incidence or severity of EAE. The observations that IL-3 is expressed in encephalitogenic T cells and myelin-specific memory T cells from MS patients (27) indicate that IL-3 may be a marker of encephalitogenic T cells, possibly due to transcriptional upregulation of the *Il3/Csf2* gene cluster, but an unlikely therapeutic target.

## Ethics Statement

The protocols used for these experiments received prior approval by the OSU Institutional Animal Care and Use Committee and were conducted in accordance with the United States Public Health Service’s Policy on Humane Care and Use of Laboratory Animals.

## Author Contributions

PL and MX designed and performed experiments, analyzed data, and assisted with writing the manuscript. WP assisted with the *in vivo* experiments. YY and AL-R designed experiments, analyzed data, and prepared the manuscript.

## Conflict of Interest Statement

The authors declare that the research was conducted in the absence of any commercial or financial relationships that could be construed as a potential conflict of interest.
